# The Effects of Acute Post Exercise Consumption of Two Cocoa-Based Beverages with Varying Flavanol Content on Indices of Muscle Recovery Following Downhill Treadmill Running

**DOI:** 10.3390/nu6010050

**Published:** 2013-12-20

**Authors:** Katelyn Peschek, Robert Pritchett, Ethan Bergman, Kelly Pritchett

**Affiliations:** Department of Nutrition, Exercise and Health Science, Central Washington University, 400 E. University Way, Ellensburg, WA 98926, USA; E-Mails: sharpk@cwu.edu (K.P.); pritcher@uga.edu (R.P.); bergmane@cwu.edu (E.B.)

**Keywords:** sports nutrition, recovery, cocoa, flavanols, antioxidants, creatine kinase

## Abstract

Dietary flavanols have been associated with reduced oxidative stress, however their efficacy in promoting recovery after exercise induced muscle damage is unclear. This study examined the effectiveness of acute consumption of cocoa-flavanols on indices of muscle recovery including: subsequent exercise performance, creatine kinase, muscle tenderness, force, and self-perceived muscle soreness. Eight endurance-trained athletes (VO_2max_ 64.4 ± 7.6 mL/kg/min) completed a downhill running protocol to induce muscle soreness, and 48-h later completed a 5-K (kilometer) time trial. Muscle recovery measurements were taken at PRE, 24 h-POST, 48 h-POST, and POST-5K. Participants consumed 1.0 g of carbohydrate per kilogram of body weight of a randomly assigned beverage (CHOC: 0 mg flavanols *vs*. CocoaCHOC: 350 mg flavanols per serving) immediately after the downhill run and again 2 h later. The same protocol was repeated three weeks later with the other beverage. An ANOVA revealed no significant difference (*p* = 0.97) between trials for 5 K completion time (CHOC 1198.3 ± 160.6 s, CocoaCHOC 1195.5 ± 148.8 s). No significant difference was found for creatine kinase (CK) levels (*p* = 0.31), or muscle soreness (*p* = 0.21) between groups over time. These findings suggest that the acute addition of cocoa flavanols to low-fat chocolate milk offer no additional recovery benefits.

## 1. Introduction

Exercise results in an increased production of free radicals, leading to cell damage and oxidative stress [1,2]. Recently, dietary flavanols with antioxidant properties have gained attention in their potential to reduce oxidative stress and aid in post exercise muscle recovery [2,3,4]. There are a couple different ways antioxidants aid in protecting the body against free radicals. Antioxidants can prevent lesser radicals from transforming to more damaging radicals, and antioxidants can convert reactive oxygen species into less active versions [5,6,7]. Although the relationship between antioxidants and free radical activity is understood, research that has examined the efficacy of antioxidant supplementation on indices of exercise recovery is controversial [2,8].

Research suggests that the dietary flavanols found in natural cocoa powder provide cardiovascular health benefits including decreasing blood pressure by improving endothelial function, and improving blood circulation [9]. It is hypothesized that an improvement in blood circulation may allow for a better delivery of nutrients and oxygen to working muscles as well as a more efficient removal of waste products that are generated by the working muscles [2,9,10]. However, it is unknown whether the ingestion of cocoa flavanols (350 mg immediately, and 350 mg again 2-h post exercise) may enhance recovery.

Athletes and coaches are constantly searching for the optimal recovery beverage. Evidence continues to emerge in support of chocolate milk, a cocoa-based beverage, as an effective recovery aid due to its optimal carbohydrate to protein ratio [11,12,13,14,15]. However, it should be noted that the cocoa found in chocolate milk has gone through the alkalization process therefore reducing the antioxidant potential [10]. Perhaps the addition of cocoa flavanols (350 mg immediately, and 2-h post exercise) could further enhance the antioxidant capabilities of low fat chocolate milk and further enhance exercise recovery. Little research is available in regards to the efficacy of natural/unprocessed cocoa as a post exercise recovery aid. Research is warranted to determine if natural/unprocessed cocoa may provide a greater improvement in recovery given the abundance of flavanols, more specifically the two subunits are catechin and epicatechin [2]. McBrier *et al*. [2] investigated the effects of a cocoa-based carbohydrate and protein beverage on muscle damage following exhaustive exercise and suggested that the cocoa-based beverage significantly (*p* = 0.03) reduced the level of perceived muscle soreness 24 to 48 h following exhaustive exercise. The authors concluded that the significant decrease in perceived muscle soreness between 24 and 48 h post exercise may be attributed to the increased levels of antioxidants in the beverage. Further research is warranted to examine the efficacy of an acute supplementation with a cocoa-based beverage (350 mg immediately, and 350 mg, again, 2-h post exercise) with varying flavanol contents as a post-exercise recovery aid. Therefore, this study has two purposes, to examine the effectiveness of an acute supplementation with natural cocoa on (1) markers of muscle recovery and perceived soreness; and (2) exercise performance during a 5 K time trial following exercise induced muscle damage.

## 2. Methods

### 2.1. Subjects

Well-trained male runners and triathletes (*n* = 8) with at least two years experience with endurance type activities, and currently training for at least six or more hours per week were recruited for this study. All subjects were between the ages of 18–44 years of age. The number of selected participants was based on an *a priori* power analysis. Based on data from Saunders *et al*. [16], for both primary dependent measures with a power of 0.80, a minimal detectable difference could be detected in means of 350 for CK, and 3 for muscle soreness based on an estimated effect size of 1.0 SD units (SD between treatments: 200 for CK, and 2 for muscle soreness, and a two-tailed alpha-level of 0.05 power analysis indicated the need for six participants). Human Subjects Review Committee (HSRC) approval was obtained from Central Washington University. All participants read and signed a written consent form approved by HSRC. Participants were excluded if they had experienced a lower extremity injury in the past six months and/or were currently taking chronic or daily doses of anti-inflammatory medication or nutritional supplements [2]. Participants who had history of a recent illness were excluded from the study.

### 2.2. VO_2max_ and Experimental Design

A randomized, single blind, cross-over design study with the participants serving as their own control was conducted. The study consisted of an exercise session followed by a 48 h rest period and a 5 K time trial. There was a 21-day washout period and then the protocol was repeated with the other treatment beverage.

Participants were required to be in the lab for an introductory visit, as well as at 3 different times for each drink (seven lab visits total). During the introductory lab visit, anthropometric measures were taken, including height, weight, and body fat percentage. Body composition was determined using a three-site skin fold measurement on the right side of the body, using standard procedures as described by Jackson and Pollock in 1985 [17]. All measures were taken by the same, trained technician, using Lange skin fold calipers (Country Technology, Inc., Gays Mills, WI, USA). Three cyclic measurements were taken per skin fold site (chest, abdomen, and thigh). The mean measurement of each site was used to calculate body density [17]. Furthermore, body density was converted to body fat percentage using the Siri formula. VO_2max_ was determined during an incremental exercise test to volitional fatigue on a treadmill (Barimill treadmill, Woodway, Waukesha, WI, USA). Participants were fitted with a heart rate (HR) monitor transmitter (Polar, Stamford, CN, USA) at the level of the sternum. Expired air was directed through a one-way valve (Hans Rudolf, Kansas City, MO, USA) and plastic tubing was connected to a metabolic cart (Parvo Medics’ TrueOne^®^ 2400, Sandy, UT, USA), which was calibrated prior to each test with a known gas composition. A 4-L precision syringe (Hans Rudolph, Kansas City, MO, USA) was utilized to calibrate the system for measurement of ventilation.

Subjects were educated on the protocol for completing three-day food records and were asked to record dietary intake during the two trials, starting the morning of the downhill run and ending on the day of the 5 K time trial, to control for any differences in dietary intake. Participants were instructed to replicate their dietary intake during both trials.

### 2.3. Downhill Running

Participants reported to the lab one week after the introductory visit. Downhill running at a −10% grade was utilized in order to induce muscle soreness [2]. Participants completed a 5-min warm-up at 0% grade on a treadmill. After the warm-up, the treadmill was declined to a −10% grade and the speed was adjusted to 70% of their VO_2max_. This intensity was maintained for 30 min.

### 2.4. 5 K Time Trial

Subjects returned to the lab 48 h after the downhill running session to perform a 5 K running time trial. Participants completed a 5-min warm-up on the treadmill at a self-selected pace. Participants were verbally encouraged to run at a maximal effort.

### 2.5. Treatments

Two beverages were examined: a cocoa-based (processed with alkali) carbohydrate protein beverage (CHOC) (0 mg of flavanols) *vs*. a carbohydrate protein beverage plus natural cocoa (CocoaCHOC) (350 mg of flavanols) in a counterbalanced and randomized manner. Following the downhill running session, subjects consumed their beverage within 1 and at 2 h into recovery [13]. Participants received the same amount of carbohydrate immediately after exercise and at 2-h post exercise (1 g carbohydrate/kg of body weight). The beverages were isovolumic for both trials (CHOC and CocoaCHOC) [11,13,14,15]. The CHOC used in this study (Darigold low-fat chocolate milk) consisted of sucrose (glucose plus fructose), cocoa processed with alkali, salt, carrageenan, vanillin, vitamin A palmitate, and vitamin D3. The CocoaCHOC used in this study (unsweetened Cocoa Via) consisted of cocoa powder (processed with alkali), maltodextrin, natural flavor, carrageenan, cocoa powder (unprocessed), salt, and soy lecithin. Nutrient content (per 240 mL) of the test drink is listed in Table 1. All drinks were poured into unmarked bottles. During the recovery period, subjects were permitted to engage in simple activities of daily living such as walking, reading, and studying. Ad libitum water consumption post exercise was allowed, however no other food was allowed until after they had consumed the second beverage at 2-h post exercise. Furthermore, the participants were instructed to consume a similar diet during periods of testing.

**Table 1:** Comparison of macronutrient content for the recovery beverages.

Total energy and macronutrient content	CHOC	CocoaCHOC
Energy (Kcals)	190	220
Carbohydrate (g)	32	37
Protein (g)	10	11
Fat (g)	3	3.5
Volume (mL)	240	240

Note: Amount of beverage ingested immediately after exercise and 2-h post exercise was based on body mass (1.0 g/kg BW/h post exercise).

### 2.6. Measurements

Blood samples for creatine kinase (CK) were obtained before the first exercise session (PRE), 24 h post the first exercise session (24 h-POST) and 48 h post the first exercise session, before the 5 K (48 h-POST), for both trials. The samples consisted of 0.025 mL of blood from the fingertip using a lancet. The blood sample was collected at the fingertip using a plasma separator tube before the beginning of the first exercise session (PRE) to determine baseline CK levels. To confirm an accurate calibration, CK controls were analyzed using low (112–176 U·L^−1^) and high (843–1095 U·L^−1^) concentrations of CK. Blood samples were analyzed for the average CK absorbance difference per minute at 340 nm using a spectrometer (Genesys 10 Series Thermo Spectronic, Rochester, NY, USA). Peak accumulation for CK levels has been indicated to occur anywhere between 12 and 24 h after exercise, with subjective measures of muscle soreness peaking at 48 h post exercise. Most studies using CK examine at 24, 48, and sometimes 72-h post exercise. For the purpose of this study, CK levels were examined at 24 and 48-h after the first exercise session based off of the literature and to maintain consistency with the subjective measures [16].

Subjective measurements of muscle soreness were assessed at PRE, 24 h-POST, 48 h-POST, and POST-5K. Self-perceived muscle soreness was assessed using a 10 cm visual analog scale [18] with anchor points “no pain at all” at the left end and “unbearable pain” at the right end. Based on McBrier *et al*. [2], muscle soreness was assessed using a lower extremity functional scale (LEFS) at PRE, 24 h-POST, 48 h-POST, and POST-5K to determine the participant’s level of difficulty with various activities of daily living [2]. The LEFS is a validated survey consisting of 20 questions. It asks the participants to rate the perceived level of difficulty if they were to perform tasks on a 0–4 scale with 0 being unable to perform the activity, and 4 having no difficulty at all [19]. Upon completion of the questionnaire, percent of muscle function can be assessed. The higher the number, the higher percent of perceived muscle function the participant has.

Muscle function was assessed in the extensors of both legs using an isokinetic dynamometer (CYBEX model #770, LUMEX Inc., Medway, MA, USA) at PRE, 24 h-POST, and 48 h-POST. Velocity was set at 0°/s in order to obtain isometric force. Participants performed four quadricep extensions on each leg, the average peak value was recorded and converted from psi to Newton meters ((psi value × 0.62) × 1.356).

Muscle tenderness was assessed using a Force Algometer (Wagner Pain Test Model FPK Algometer) [20]. Measurements were made at the rectus femoris (RF) and vastus medialis oblique (VMO). All measurements were reported in kilograms (kg). Force was applied via the probe through a 1-cm diameter head until the participant indicated pain or discomfort. At this point the force value (kg) was recorded. Muscle tenderness was measured at PRE, 24 h-POST, 48 h-POST, and POST-5K.

### 2.7. Statistical Analysis

Mean values ± standard deviation (SD) for heart rate, rate of perceived exertion, and perceived muscle soreness were computed for each treatment. Participants’ three-day diet records were analyzed using a computer software program (ESHA Food Processor, version 10.6, Salem, OR, USA) to examine total kcal, carbohydrate, protein, and fat consumption. Data from the two trials were compared using a two-factor (treatment × time) repeated–measures analysis of variance (ANOVA). A one-way Repeated Measures (RM) ANOVA was used to compare CK, muscle soreness (VAS, LEFS), isometric force, muscle tenderness, and performance between trials. Greenhouse-Geisser corrections were conducted for unequal variance data. Statistical Package for the Social Sciences for Windows software version 16.0 (Statistical Package for Social Sciences (SPSS) Inc., Chicago, IL, USA) was used for all statistical analyses. All data were reported as means ± standard deviation. Statistical significance was set at α < 0.05 for all analyses.

## 3. Results

Descriptive characteristics reported as means ± SD for each participant are as follows: age (years): 24.6 ± 5.6, height (cm): 182.1 ± 6.3, weight (kg): 73.4 ± 7.0, and percent body fat: 13.7 ± 5.1. According to the dietary analysis there were no significant differences in macronutrient composition (kilocalories, CHO, PRO, and FAT) between trials (Table 2).

**Table 2:** Average daily dietary intake for each trial (*n* = 8).

Total energy and macronutrient content	CHOC	CocoaCHOC
Energy (kcals)	2632.3 ± 1205.1	3261.7 ± 1743.4
Carbohydrate (g)	310.2 ± 144.9	411.5 ± 223.6
Protein (g)	103.2 ± 56.9	106.4 ± 63.3
Fat (g)	99.6 ± 55.2	127.5 ± 75.5

Note: Values do not include the macronutrients in the recovery beverages. No significant differences were observed between trials. Data reported are means ± SD.

Performance was measured by the amount of time it took for the participants to complete a 5 K time trial using a stopwatch. There was no significant difference (*p* = 0.97) between trials for 5 K times (CHOC 1198.3 ± 160.6 s, CocoaCHOC 1195.5 ± 148.8 s). To test for a repeated bout effect, the test of the treatment effect suggested that there was no significant difference (*p* = 0.48) between the first and the second period for each individual, and there was no significant difference (*p* = 0.71) for the period effect.

There was no significant difference (*p* = 0.33) found in regards to isometric force overtime between trials (Table 3). When looking at percent change overtime in the CHOC group, there was a 5% and 2.3% decrease from PRE to 24 h-POST and a 5.10% and 5.7% decrease from PRE to 48 h-POST in the right and left legs, respectively. When looking at percent change overtime in the CocoaCHOC group, there was a 10.3% and 4.3% decrease from PRE to 24 h-POST, and a 22.2% and 11.8% decrease from PRE to 48 h-POST in the right and left legs, respectively.

**Table 3:** Isometric force over time between treatments (Nm) (*n* = 8).

Time	CHOC (R-leg)	CocoaCHOC (R-leg)	CHOC (L-leg)	CocoaCHOC (L-leg)
PRE	148.9 ± 32.9	166.8 ± 42.7	153.8 ± 40.6	180.4 ± 56.4
24-h-Post	141.5 ± 21.6	149.6 ± 26.2	150.3 ± 38.8	140.4 ± 18.5
48-h-Post	141.4 ± 15.3	159.6 ± 31.8	145.1 ± 29.7	159.1 ± 33.6

Note: Nm, Newton meters; CHOC, chocolate milk; R-leg, right leg; L-leg, left leg; CocoaCHOC, cocoa beverage. Data reported as means ± SD.

**Figure 1:**
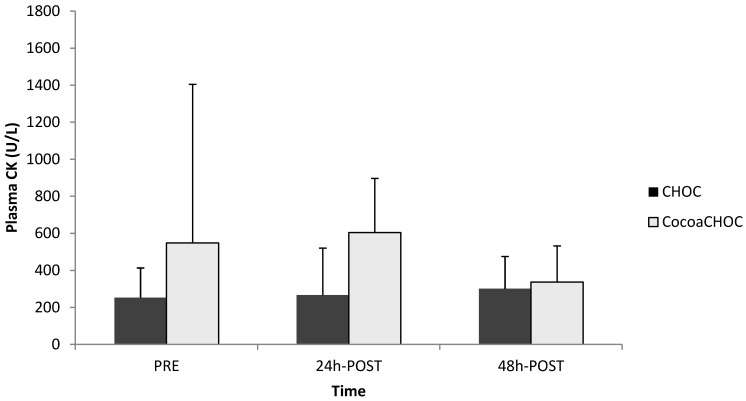
Creatine kinase (CK) levels for each trial (CKPRE, CK 24 h-POST, and CK 48 h-POST). Note: CHOC, chocolate milk; CocoaCHOC, cocoa beverage; CK, creatine kinase; U/L, units/liter.

The results for CK levels are displayed in Figure 1. There was no main effect (treatment × time) (*p* = 0.31) for CK observed within groups. A one-way ANOVA revealed no significant difference between groups for CKPRE (*p* = 0.35) (CHOC 252.97 ± 160.44 U/L, CocoaCHOC 548.37 ± 856.84 U/L), for CK 24 h-POST (*p* = 0.77) (CHOC 266.78 ± 254.38 U/L, CocoaCHOC 604.39 ± 291.99 U/L), and for CK 48 h-POST (*p* = 0.27) (CHOC 301.16 ± 174.27 U/L, CocoaCHOC 337.41 ± 195.04 U/L). There was no significant change (*p* = 0.86) from CKPRE to CK 48 h-POST, as well as no significant change from CKPRE to CK 24 h-POST observed in both trials.

**Figure 2:**
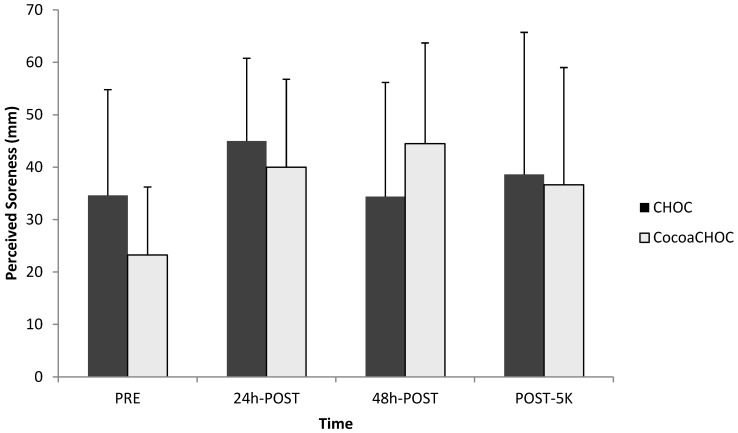
Mean ± SD perceived muscle soreness using the visual analog scale (VAS) at PRE, 24 h-POST, 48 h-POST, and POST-5K for each treatment beverage (CHOC, CocoaCHOC). Note: CHOC, chocolate milk; CocoaCHOC, cocoa beverage; mm, millimeters.

Perceived muscle soreness using a VAS, as well as a LEFS, was examined at PRE, 24 h-POST, 48 h-POST, and POST-5K. No interaction (treatment × time) (*p* = 0.23) for muscle soreness using the VAS was observed (Figure 2).

**Figure 3:**
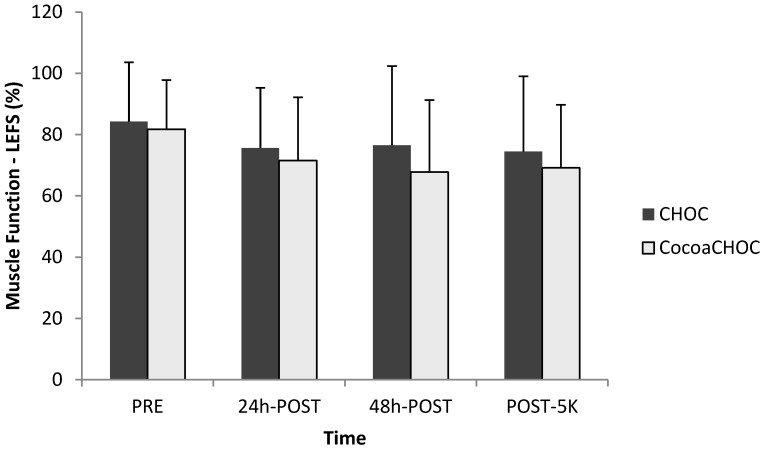
Mean ± SD percent muscle function (LEFS) at PRE, 24 h-POST, 48 h-POST, and POST-5K between treatment beverages. Note: CHOC, chocolate milk; CocoaCHOC, cocoa beverage; LEFS, lower extremity functional scale.

No interaction-time (treatment × time) for muscle soreness using the LEFS was observed. The results for LEFS are shown in Figure 3.

A repeated measures ANOVA indicated no significant interaction (treatment × time) for muscle tenderness of the RF (*p* = 0.95) as well as for muscle tenderness of the VMO (*p* = 0.07) between the two treatments (Table 4). No significant difference (*p* = 0.08) was seen in change from VMO PRE to 24 h-POST and VMO 48 h-POST to POST-5K (*p* = 0.08).

**Table 4:** Mean muscle tenderness scores of the rectus femoris (RF) and vastus medialis oblique (VMO) between treatment beverages over time (*n* = 8).

Timing	CHOC (kg)	CocoaCHOC (kg)
RF PRE	5.4 ± 1.8	5.9 ± 1.2
RF 24 h-POST	5.7 ± 1.6	6.3 ± 1.2
RF 48 h-POST	5.5 ± 1.7	6.0 ± 1.6
RF POST-5K	5.3 ± 1.9	6.1 ± 0.8
VMO PRE	4.4 ± 1.7	4.8 ± 1.5
VMO 24 h-POST	4.4 ± 1.4	5.6 ± 0.9
VMO 48 h-POST	4.6 ± 1.9	4.4 ± 1.6
VMO POST-5K	4.3 ± 1.8	4.5 ± 1.6

Note: RF, rectus femoris; VMO, vastus medialis oblique; CHOC, chocolate milk; CocoaCHOC, cocoa beverage; kg, kilograms; SD, standard deviation. Values are Mean + SD.

## 4. Discussion

This study examined the effects of CHOC *vs*. CocoaCHOC on indices of muscle damage including perceived soreness (VAS and LEFS), CK, and muscle tenderness. Exercise performance was assessed using a 5 K time trial and isometric force to compare the efficacy of the treatment beverages. The primary findings of our study suggest that the acute consumption of cocoa-flavanols added to a cocoa-based recovery beverage provide no additional benefits on markers of muscle recovery and perceived soreness. Furthermore, there were no differences in subsequent exercise performance following exercise induced muscle damage between the two treatments.

Although not significant, an increase in perceived muscle soreness from baseline at either 24 h or 48 h after the downhill exercise protocol indicated that the downhill running protocol was effective in inducing delayed onset muscle soreness (DOMS). Similar to the findings of McBrier *et al*. [2] no change (*p* = 0.27) was observed from CKPRE to CK 48 h-POST in both trials. Failure to detect a significant increase in CK levels may be due to the elevated levels seen at baseline, which may be attributed to the chronic level of muscle damage seen in trained athletes. In addition to blood parameters of muscle damage, muscle soreness was examined at PRE, 24 h-POST, 48 h-POST, and POST-5K. Several studies have examined multiple measures of recovery in order to enhance the study design [2,12,13,21]. Subjective measures of muscle soreness using the visual analog scale (VAS) indicated no significant difference in perceived soreness for both treatment groups.

The current study utilized another form of evaluating perceived soreness, the lower extremity functional scale (LEFS). A primary benefit of the LEFS is that it examines functionality of multiple activities of daily living in one questionnaire [2,19]. Therefore, as seen in this study, a decline in scores would indicate a decrease in perceived muscle function. Subjective measures of muscle soreness using the LEFS suggested that the percent of muscle function decreased over time following the downhill running protocol in both treatment groups.

Other studies [2] examining cocoa flavanols on exercise recovery have not examined subsequent exercise performance. From a practical perspective, exercise performance should be assessed when determining if a recovery treatment is effective. This study examined differences in exercise performance 48 h after inducing DOMS when CHOC or CocoaCHOC was consumed during the recovery period. The beverages were similar in nutritional composition (kcals, CHO, protein, and fat) except for the flavanol content (CHOC: 0 mg of flavanols; CocoaCHOC: 350 mg of flavanols per serving), however no significant differences were discovered in regards to 5 K completion time indicating that the acute consumption of additional cocoa flavanols to a recovery beverage offer no further benefits. It should also be noted that the current study utilized a time trial (similar to a race situation), while other studies examining recovery use time to exhaustion [13]. Furthermore, in contrast to other research examining the efficacy of dietary flavanols, this study implemented an acute dose of dietary flavanols. Other dietary flavanol research studies have implemented a five-day to two-month-long loading phase [4,20,22].

Although there has been a significant volume of research completed on DOMS, exercise performance, and post-exercise antioxidant recovery beverages, there are several inconsistencies in regards to the methods of research employed as well as varying results [2]. Variations of methodology include the timing of supplementation, dosage, and measurements of muscle soreness. Some studies have examined acute supplementation pre exercise [20,23], post exercise [2], or before and after exercise [22], whereas the majority of antioxidant and flavanol studies have examined long-term supplementation [4,24]. Nishizawa *et al*. [4] examined the effect of flavanol-rich lychee fruit extract for two-months during intense training on inflammatory status and tissue damage of young athletes. Using a timed 5000 m track race they found no significant difference between the two groups (data not shown) [4]. Connolly *et al*. [20] examined the efficacy of 12 fl oz twice daily of a tart cherry juice blend for eight days and suggested that pain was significantly less (*p* *=* 0.01) in the cherry juice group when compared to the placebo group [20]. Finally, in comparison to the current study, the majority of the literature examining dietary flavanols (tart cherry juice, *etc*.) has examined a longer supplementation period. However, the rationale for providing a long supplementation period is not provided in the literature.

In regards to dosage, the cocoa beverage utilized in the current study provided 350 mg of flavanols per beverage. As referred to previously, participants consumed the CocoaCHOC beverage immediately post the downhill run and again 2 h later, therefore they received a total of 700 mg of flavanols. The flavanol content in the cocoa-based CHO/PRO beverage of the McBrier study is unknown [2]. Other studies have utilized a variety of flavanol dosing techniques. Some examples include: 50 mg per day for two months [4], 560 mg for five days prior to exercise, the day of exercise, and again 48 h post exercise [22], and 600 mg two times per day for eight days [20].

This study is not without limitations. This study only used male participants in order to control for any physiological variables between genders. In addition, based on other studies [4,20,22,24], it may be more beneficial to have subjects load the supplement, rather than administering it acutely, in order for the flavanols to have enough time to aid in tissue repair. However, other studies don’t document a rational or proposed mechanism for including a loading phase. Lastly, there may have been a “learned effect” from this study, meaning that subjects may be more accustomed running downhill during the second trial compared to the first trial. However, we based our study on other current literature that suggested that a three-week washout period [2] and counterbalanced, crossover design should eliminate the issue of the repeated bout effect. Furthermore, subjects were familiarized to the treadmill during the VO_2max_ testing.

This study aimed to examine the efficacy of cocoa-flavanols on muscle recovery and exercise performance following exercise induced muscle damage. The study demonstrated that acute post exercise consumption of a beverage consisting of 350 mg of cocoa-flavanols post exercise was not significantly different than consuming chocolate milk, post exercise, in regards to muscle recovery and exercise performance. Plasma CK levels and subjective measurements of muscle soreness were similar at 24 h-POST, 48 h-POST, and POST-5K for both treatments. Furthermore, 5 K performance was not significantly different between trials. Perhaps if this study had implemented a loading phase similar to other studies examining dietary flavanols and exercise recovery, a difference in indices of muscle recovery may have been detected. Future research should explore whether a dose response exists, or if employing a loading phase with cocoa flavanols prior to inducing muscle damage is effective in improving muscle recovery.

## 5. Conclusions

These findings suggest that the addition of cocoa-flavanols to a post exercise recovery beverage offers no additional benefits when compared to consuming chocolate milk as a recovery aid. The administration of two recovery beverages closely matched for Kcals, CHO, PRO, and fat content resulted in similar findings. Additional studies are warranted to examine the potential advantage of cocoa flavanols on indices of exercise recovery. More specifically, future studies should examine the consumption of cocoa flavanols for longer periods of time rather than acutely.
